# Factors Increasing Vulnerability to Health Effects before, during and after Floods

**DOI:** 10.3390/ijerph10127015

**Published:** 2013-12-11

**Authors:** Dianne Lowe, Kristie L. Ebi, Bertil Forsberg

**Affiliations:** 1 Department of Public Health & Clinical Medicine, Occupational and Environmental Medicine, Umeå University, SE-90187 Umeå, Sweden; E-Mails: krisebi@essllc.org (K.L.E.); bertil.forsberg@envmed.umu.se (B.F.); 2 Centre for Health Communication and Participation, School of Public Health & Human Biosciences, La Trobe University, Victoria 3086, Australia

**Keywords:** vulnerability, floods, risk factors, humans, health

## Abstract

Identifying the risk factors for morbidity and mortality effects pre-, during and post-flood may aid the appropriate targeting of flood-related adverse health prevention strategies. We conducted a systematic PubMed search to identify studies examining risk factors for health effects of precipitation-related floods, among Organisation for Economic Co-Operation and Development (OECD) member countries. Research identifying flood-related morbidity and mortality risk factors is limited and primarily examines demographic characteristics such as age and gender. During floods, females, elderly and children appear to be at greater risk of psychological and physical health effects, while males between 10 to 29 years may be at greater risk of mortality. Post-flood, those over 65 years and males are at increased risk of physical health effects, while females appear at greater risk of psychological health effects. Other risk factors include previous flood experiences, greater flood depth or flood trauma, existing illnesses, medication interruption, and low education or socio-economic status. Tailoring messages to high-risk groups may increase their effectiveness. Target populations differ for morbidity and mortality effects, and differ pre-, during, and post-flood. Additional research is required to identify the risk factors associated with pre- and post-flood mortality and post-flood morbidity, preferably using prospective cohort studies.

## 1. Introduction

Floods are amongst the most significant “natural disasters” in terms of the number of persons affected [1]. Flash floods result in the highest average mortality per event (defined as the number of fatalities divided by the number of persons affected) [1]. The extent to which a flood causes impacts is determined not just by the magnitude of the flood, but also by human and societal choices related to infrastructure, behavior, and other factors. For this reason, the disaster risk management community prefers to not use the term natural disaster when describing floods, as it has a connotation that a disaster cannot be avoided. Health effects of floods may include hospitalization or emergency department visits, psychological effects, physiological injury, illness or infection or mortality [2]. 

The risk that a flooding event will be a disaster is a function of three factors: the hazard associated with the flood; the human and natural systems exposed to the floodwaters; and the vulnerability of these systems to flooding [3]. The frequency and intensity of extreme precipitation events are likely to increase with climate change [4], with the number of people at risk of being exposed to flooding effects likely to increase [5]. It is anticipated that extreme precipitation events will increase in southern Asia; during winter in northern Europe; in the United Kingdom (UK) during winter, spring, and autumn; and over the southern and central United States (US). It is anticipated such events will decrease during summer in the south of Europe; in Canadian prairies and southern Australia [3]. Extreme precipitation events that were previously rare, occurring once in twenty years, are projected to become more frequent in the future [3,6,7]. Impacts will continue to arise not just because of changes in precipitation intensity, but also because of more people living in harms’ way. Localised human activities, including populating flood prone areas, environmental transformations (such as railway, drinking water and sewage systems), river modifications and, ironically, flood management schemes, can increase the incidence and severity of flood events [8,9,10].

Exposure to being flooded is influenced by environmental, political and commercial activities, as well as geographic proximity [11]. Vulnerability to being flooded appears to be greater in individuals with pre-existing social vulnerability, particularly related to socio-economic, demographic and health factors. Flood impacts are magnified by lack of awareness, limited mobility or physical capacity, fewer resources to protect, insure or repair property and limited social networks [12]. In the UK, there is evidence of significant inequalities in patterns of exposure to floods and the experience of flood impacts in relation to deprivation and poverty, and in terms of age and gender [13].

The broader (non-health related) impacts of exposure to floods include widespread damage to property and possessions, rescue or immediate assistance needs during floods, homelessness, displaced and evacuated households and economic consequences. These broader impacts are unequally distributed amongst populations [14]. Further, there is a suggestion that over time individual responses to flood events have changed from monitoring and implementing adaptive strategies (e.g., preemptively moving belongings to upper levels during flood conducive weather patterns), to dependency on potentially fallible river modification and flood management schemes, followed by diminished capacity to cope [10]. 

While there is research identifying the health effects of floods [5], and the characteristics of floods associated with health effects, little is known about factors that increase individual vulnerability to these health effects among those flooded (e.g., given that one is flooded, what factors increase vulnerability?). Undeniably the experience of health effects is directly related to being exposed to floodwater, however, not all individuals who are exposed to floodwater experience health effects. Those unexposed directly to floodwater but living in the vicinity of floods can also experience health effects while preparing to evacuate or experiencing relocation from home. 

In this review, we focus on member countries of the Organisation for Economic Co-Operation and Development (OECD) as they are relatively comparable, unlike low-income countries. There appears to be a large difference between the overall economic impacts of flooding in high, middle and low income countries in terms of the percentage of gross domestic product (GDP) spent on flood management. For all weather related disasters, expenditure between 2001 to 2006 was 0.1% of average high-income countries GDP, 0.3% of low-income countries and 1.0% of middle-income. The differences can be attributed to the value of the infrastructure with middle-income countries having the largest burden due to expanding asset bases [3]. 

**Figure 1:**
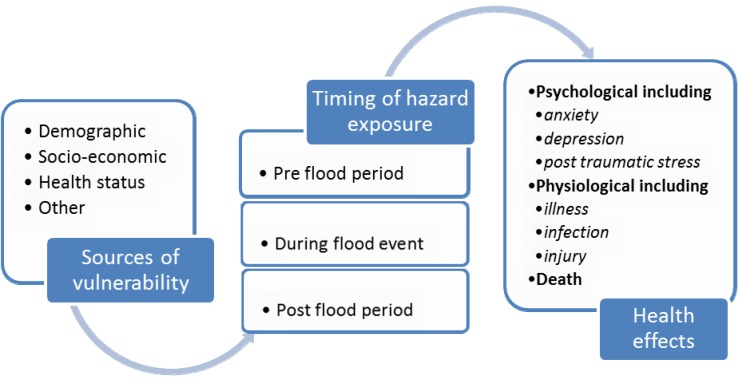
Factors increasing vulnerability to health effects of floods before, during and after flooding.

The risk factors (individual, demographic or socio-economic characteristics) increasing vulnerability to health effects of floods may differ based on the timing of exposure, whether before, during or after flood events (see Figure 1). We sought to systematically examine the research identifying risk factors increasing vulnerability to flood-related adverse human health effects before, during and after (short and longer term follow-up) flood events, within OECD countries. Identifying risk factors that increase vulnerability to health effects may aid the appropriate targeting of health prevention strategies [15]. Enabling emergency response and health systems to prepare for and respond to flood disasters by identifying and targeting individuals susceptible to health effects of floods requires identification of these risk factors prior to developing emergency medical systems to enhance disaster response capacity.

**Figure 2:**
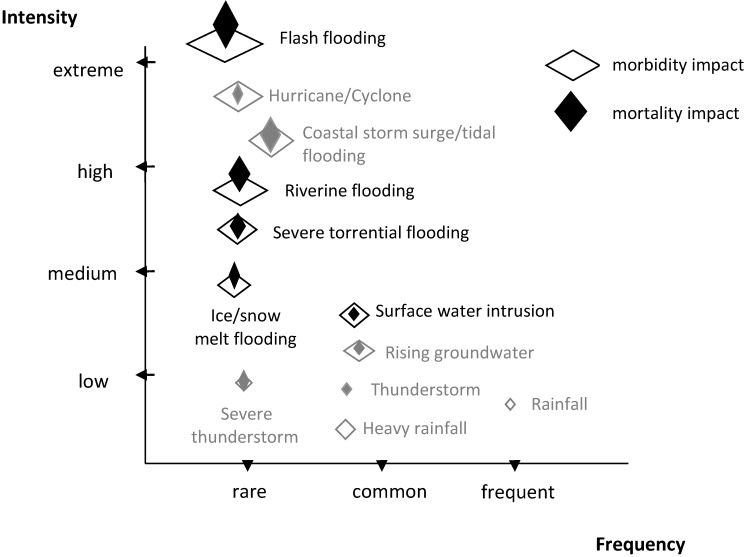
Severity, frequency and impact of extreme hydrological weather events. We focused on extreme precipitation-related flood events that are sometimes compounded by snow and ice melt e.g., severe torrential rain, flash and riverine flooding, (bolded) and excluded minor flooding events and those accompanying extreme wind or tides (greyed). The diamond size illustrates the typical magnitude of the morbidity or mortality impact [1,16,17].

While there are a number of extreme water-related events that have health effects, we focus on extreme and/or prolonged rainfall events (as bolded in Figure 2), sometimes compounded by quick snow melts, which contribute to urban floods, rural ponding, pluvial river, and flash floods, rather than cyclones, coastal storms, or tidal flooding (presented in grey text in Figure 2). When floods are ranked by fatalities per flood event, it is evident that flash floods (especially those from dam failures) and floods associated with tropical storms are the flood types most typically associated with large numbers of fatalities [16]. 

## 2. Experimental Section

To identify the factors that increase vulnerability to the human health effects from floods, we searched PubMed for:

Flood-related terms:

- “floods”[MeSH Terms] OR
- “floods”[All Fields] OR
- “flood”[All Fields]
- AND risk factor terms
- “risk factors”[MeSH Terms] OR
- “risk”[All Fields] AND "factors”[All Fields] OR
- “risk factors”[All Fields]
- AND in humans
- “humans”[MeSH Terms]

We additionally searched the internet and the citation listings of relevant publications. We included all study designs (case series, case reports, cohort studies, controlled before and after studies) that examined relationships between individual risk factors that increase vulnerability to extreme rainfall associated flood-health effects. The focus of this review was an OECD context, as these countries are comparatively similar with respect to management and experience of floods. Studies that examined the impact of coastal floods were excluded from this review. 

Studies were included if they fit all the following criteria: (a) examined risk factors, (b) human health effects from flooding, (c) extreme rainfall related flooding, and in (d) in an OECD country. To understand the types of flood-health effects and identify possible vulnerability factors, we specifically sought to identify and categorise the included studies to four potential research questions:

1. What are the demographic, socioeconomic, health status, or other factors associated with increased risk of morbidity or mortality, among flooded populations?
2. What are the health effects of floods when compared to non-flooded groups?
3. What are the characteristics of individuals who experienced flood-related morbidity or mortality?
  - With reference to a source population.
  - Without reference to source population.

Studies that answer questions 1 and 3a are the primary focus of this review. Studies answering the other questions were included as supportive material. 

For all included studies, we extracted data on the study characteristics including design, type of flood event, risk factors, health outcomes, methods, and findings. We categorised findings based on the likely timing of the occurrence of the health outcome, e.g., before, during or after the flood event. Findings from studies examining risk factors for which it was apparent that the outcome preceded and was conceivably related to flood event (such as heart attacks while sandbagging) were categorised as “pre- flood”. Findings from studies examining risk factors for which it was apparent that the outcomes were delayed and were conceivably related to the flooding event (such as respiratory illnesses or psychological effects) were categorised as “post-flood”. Post-flood findings were separated into two further categories short-term (up to three months immediately post-flood) and longer term (three months or longer). The remainder of the findings were categorised as during-flood and include study findings for which it is apparent that the outcomes were the result of and occurred during the flood event itself or where timing was not reported. 

## 3. Results

The PubMed search (May 2013) identified 286 records, of which 45 were obtained in full text. Thirteen of these studies were included after reviewing full texts. The remaining included studies were identified through bibliography “snowballing” and Google searches. In total, we included 38 studies in this review. Of the excluded studies, reasons for exclusion were the study was not related to flooding or it focused on a cyclone, hurricane, coastal or other non-precipitation related flooding event; the study examined non-health effects; or the setting was a non-OECD country. 

Of the included studies, 17 studies identify the risk factors associated with health effects amongst flooded populations (*i.e.*, research question 1) [18,19,20,21,22,23,24,25,26,27,28,29,30,31,32,33,34]. The characteristics and findings from these studies are presented in Table A1. A small number of studies (4) identify characteristics increasing the risk of flood health effects (*i.e.*, research question 3a) [16,17,35,36], but these studies do not distinguish between the characteristics that increase risk of exposure to being flooded and the characteristics that increase risk of health effects once flooded. The characteristics and findings from these studies are presented in Table A2. 

A total of 16 studies examined the health effects that can be attributed to floods, by comparing the health of the flooded with those who were not flooded (*i.e.*, research question 2) [19,22,25,32,33,37,38,39,40,41,42,43,44,45,46,47]. While some of these studies measured demographics, they typically treat those characteristics as confounders of the flood-health relationship, for example, by adjusting for differences between the groups in terms of age and gender. Although this is valuable information, these studies do not examine age and gender differences between those who experience health effects compared to those who do not, amongst those flooded. Despite this, we included these studies, to identify the health effects attributed to floods and to identify where there may be gaps in terms of knowing which individual risk factors contribute to health effects among flooded populations. The characteristics and findings from these studies are presented in Table A3.

Finally, six studies describe characteristics of those who suffered health effects at the time of floods (*i.e.*, research question 3b) [1,48,49,50,51,52], but it is unclear if the frequency of these characteristics are more common among those flooded than for the source population or if the health effects can be attributed to the floods. However, they may identify suggested areas for future research. The characteristics and findings from these studies are presented in Table A4.

Of the included studies, five address more than one of the research questions and are therefore represented in more than one table [19,22,25,32,33]. 

**Figure 3:**
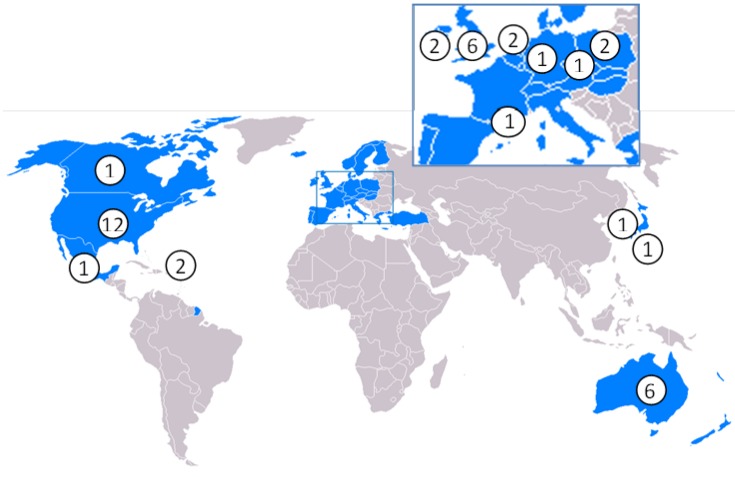
Number of studies identified for each OECD country. *NB* There were studies of Europe and US [48] and worldwide [1] that are not illustrated.

Of the OECD countries (see Figure 3), the studies were primarily conducted in the United States (US) [16,17,21,28,29,31,32,44,47,49,52], United Kingdom (UK) [22,23,37,38,39,40], parts of Europe [18,19,24,26,27,43], and Australia [25,35,36,42,45,50]. There are single studies for Korea [30], Japan [41], Canada [51], and Mexico [46]. 

Seven studies are cohort design [21,30,31,32,33,37,44], four are case control studies [19,20,39,43], and three are before and after control studies [25,38,42]. The remainder includes cross-sectional surveys, case series, and observational, archival or historical reports. 

**Figure 4:**
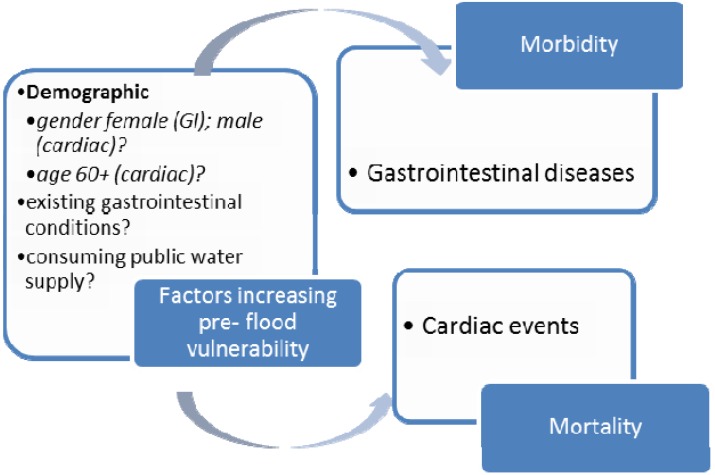
Risk factors increasing vulnerability to health effects before flood events.

**Figure 5:**
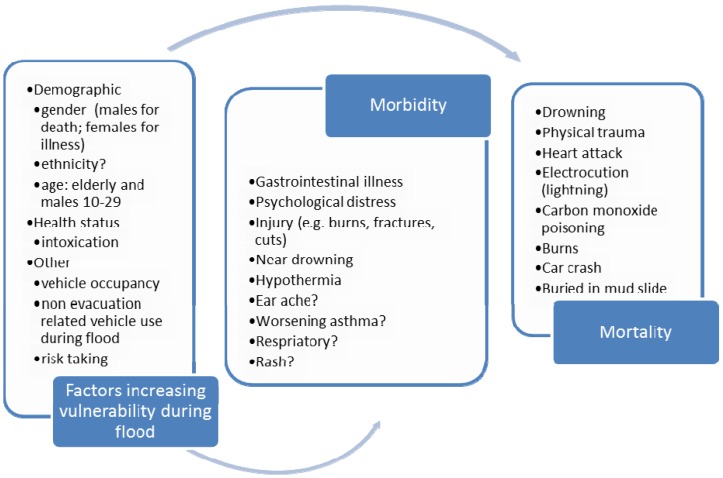
Factors increasing vulnerability to health effects during-flood events.

**Figure 6:**
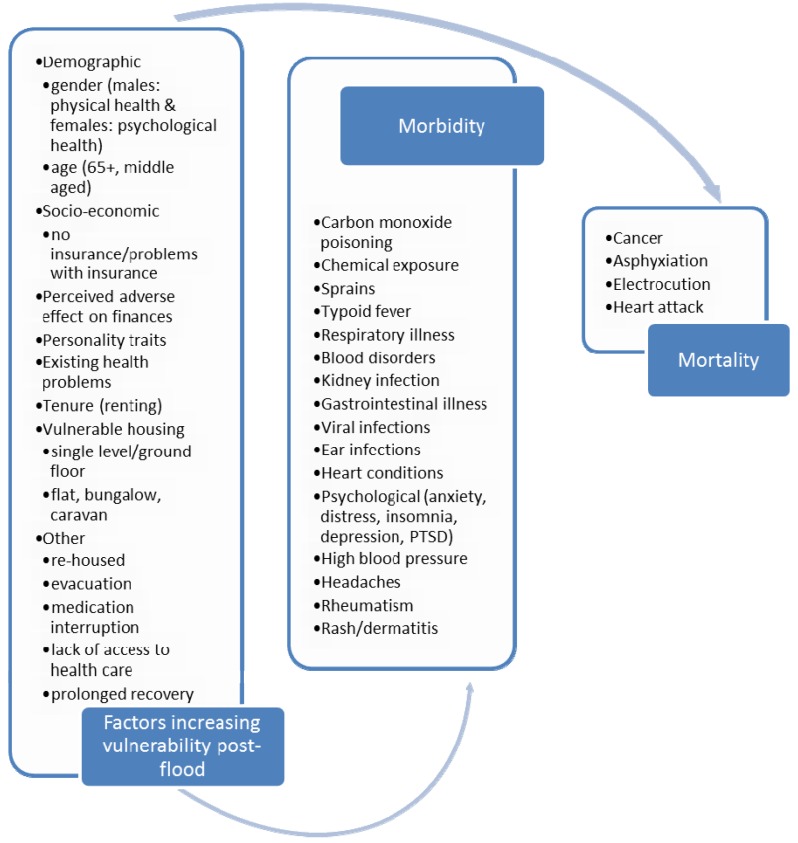
Factors increasing vulnerability to health effects post-flood events.

For each study, the extracted findings were collated in an overview figure for each of the time points, (*i.e.*, before, during or after the flood event) (see Figure 4, Figure 5 and Figure 6). Within these figures, the first cell identifies the factors associated with vulnerability, the second cell identifies the range of morbidity outcomes and the third cell, the range of mortality outcomes, with the arrows suggestive of causal pathway connections. 

Findings from studies that examined questions 1 and 3a were incorporated into tables (see Table A5 and Table A6) that illustrate the factors associated with statistically significant increasing risk (risk factors), or significantly decreasing risk (protective factors), and factors that were not significantly associated with the health outcomes. Table A5 illustrates during-flood factors, and Table A6 illustrates post-flood factors. 

### 3.1. Health Effects Observed Pre-Flood

Three of the included studies examined flood-related mortality that preceded floods [19,48,49]. Two studies found no mortality [19,48]. The third study, a case series of US flood-attributed deaths during 1986, observed that three of the 24 flood-related deaths preceded the flood event (deaths were attributed to heart attacks while relocating furniture or sandbagging) [49] (see Figure 4). 

None of the identified studies examined occurrences of pre-flood morbidity. However, one study found a number of cases of gastrointestinal illness (GI) preceded the flood event [51] (see Figure 4). Genetic testing of the gastrointestinal strain suggests that these cases were related to the flood, and samples of the water supply taken prior to the flood confirm this, as they contain small amounts of the same pathogen. The authors suggest some runoff may have entered the water supply up to a month before the floods, during heavy rainfall (see Figure 4).

### 3.2. Pre-Flood Vulnerability Factors

#### 3.2.1. Mortality

One US case series noted that overall, 17 of the 24 flood-attributed deaths were male, however it did not specifically report any factors that may increase vulnerability for the three observed pre-flood deaths [49].

#### 3.2.2. Morbidity

No study assessed pre-flood morbidity risk factors among flooded populations. The authors of the study that observed pre-flood morbidity did not specifically focus on the subgroup exposed prior to the flood, however, they did note that overall, 57% of all cases of GI illness were female, the median age 29 years and the majority had consumed public water supply [51].

### 3.3. Health Effects Observed during Flood

Studies from Europe, US and Australia reported during-flood mortalities; the vast majority were attributed to drowning [16,19,20,35,36,48,49,52]. Mortality was also attributed to: trauma, injury, heart attack, electrocution, burns, carbon monoxide poisoning and car crash (see Figure 5) [19,35,36,48,49,52]. 

Studies from France [19], England and Wales [22], Germany [18,34], and the US [22], detailed during-flood-event morbidity effects, including: physical injury [18,19], gastrointestinal illness [21], diarrhea [18], and psychological distress [22,34] (see Figure 5).

### 3.4. During-Flood Vulnerability Factors

#### 3.4.1. Mortality

##### Gender and Age

Only one case-control study, in Puerto Rico, used a flood-exposed control group to examine if gender, or age, influenced the risk of mortality relative to those who were not flooded [20]; neither altered mortality risk [20]. 

Similarly, crude death rates from storms and floods, in the US from 1979 to 2004 (using 2000 US census data), indicate that deaths did not vary by gender [17]. The majority of other studies, report the proportion of the sample who died by gender [16,35,36,48,49]. For these studies, if we assume an equal gender distribution in the flood-affected populations males are at a much higher risk of dying during floods, particularly males between 35–54 years in Australia [36], and males between the 10 and 29 years in US [16]. However, gender was unknown for 49% of deaths in one study [16] and 16.2% in another [48]. Although not consistently reported, there may be a trend towards females in the oldest age group being vulnerable [36], and cultural factors may drive these differences [1]. 

Studies reporting during-flood mortality effects do not give a clear picture as to which age groups are at increased risk. Even where they refer back to the source population, they do not distinguish whether an age group was more commonly exposed to the flood or more affected if exposed. However, in the US (1979 to 2004), crude death rates attributed to floods (using 2000 US census data), were highest among those over 55 years [17]. In addition to older age groups (those over 60 [16] and over 70 [35]) being at higher risk, studies in the US (compared to the census population [16]) and in Australia (without comparison group or comparison to census data [35]) found those between 10 and 29 years were also vulnerable to flood-related deaths. The US study found those between 30 and 59 years were less likely to experience flood-related fatalities [16]; however age was not reported for 63% of deaths. In contrast, a study examining fatalities in Australia, using death rates per 1,000,000 population, found increases among those between 35 and 54 years, those 59 years or older and those younger than 25 [36]. 

##### Ethnicity

Crude death rates did not vary for ethnicity, among 2,741 deaths associated with storms and floods from natural events, in the US from 1979 to 2004 [17]. In another study, while the authors assert that there are complex interrelations between cause of death and ethnicity (among other factors), no data were presented on ethnicity [48]. 

##### Other Factors

There is some suggestion that the numbers of flood deaths are primarily due to unnecessary risky behavior. Mortality risk factors in terms of activity and blood alcohol levels deserve further research [20,48]. 

Only one study (a 1992 Puerto Rican case-control) used a flood-exposed control group to examine factors related to flood mortality [20]. Vehicle occupancy elevated mortality significantly [OR: 15.9 (95% CI: 3.5–144)] [20]. It is worth noting that the control group had significantly lower income than census details of the exposed population and may not be representative of the exposed population ownership of cars. Also, blood alcohol content was positive for 12 out of 16 adult deaths; of these, five people had a blood alcohol content that exceeded 0.1% [20]. In the same study, use of a vehicle to evacuate a flooded area was protective; using the vehicle for other reasons increased the risk of mortality [20]. Vehicle use was implicated in 48.5% of 73 fatalities from flood events from 1997 to 2008 in Australia and 26.5% were attributed to inappropriate or high-risk behaviour during floods [35]. Similarly, 43% of the known drowning deaths during flood events in the US, between 1969 and 1981, were car related, the remainder were in homes, at campsites or when persons were crossing bridges and streams as pedestrians [52]. The circumstances around death were known in 64% of cases of flood-deaths between 1959 to 2005 in the US. Of these, 63% occurred in vehicles, 19% occurred on or in permanent structures, outside or alongside the flood (*i.e.*, accidental), and 9% were inside flood-water (among those over 12 years, 43% walked through floodwaters to evacuate or reach a car or house, and 16% entered floodwater to help others) [16]. 

#### 3.4.2. Morbidity

Four identified studies examined the factors associated with increased risk of morbidity, amongst those flooded, during impact [18,19,21,34]. 

Among those flooded during 2002 in Germany, those over 60 years were at increased risk of reporting the psychological and physical consequences of floods as “very bad” [34]. In this study, tenure (renting or owning home), gender, and location were not significant predictors of psychological or physical consequences [34]. As the survey was conducted retrospectively, three years post-flood, responses may be subject to recall bias. 

A study in the US identified the factors associated with increased rates of gastrointestinal illness post-flood, but not separately for the during-flood period [21]. However, this study suggests gastrointestinal symptom episodes were 1.29 times higher during the flood than during the follow-up period (95 % CI: 1.06, 1.58) [21]. A survey, of those flooded in Germany 2002, found exposures associated with the onset of diarrhea were skin contact with floodwater, being female, and water supply from a private pond [18]. In the same study, the only independent risk factor identified for injuries was skin contact with floodwater [18]. A study in France observed that the ages of the flooded subgroup who were injured did not differ from the whole flooded population [19]. 

### 3.5. Health Effects Observed Short-Term (up to 3 Months) Post-Flood

Four included studies examined short-term, post-flood, mortality in France [19], Europe and US [48,49] and Canada [51]. One study observed no incidence of death short-term post-flood [19]. Deaths related to post-flood clean-up included heart attacks and vehicle-related drowning [48,49]. Six people died following an *E. coli* outbreak in Canada attributed to polluted water entering the public water supply during flooding [51] (see Figure 6).

Six included studies examined morbidity effects in the three months post-flood [19,21,24,41,50,51]. Morbidity health effects included: carbon monoxide poisonings; chemical exposures and sprains; and typhoid fever cases, confirmed by culture, that were suspected to be flood-related, however these cases were not from the same region [19]. Other health problems identified included disruption of medication in the context of the elderly and those chronically ill [41], psychological [19], respiratory [19] and gastrointestinal illness (GI) [21,24,50,51], and specific infections (leptospirosis [50] and Tahyna virus [24]) (see Figure 6). 

### 3.6. Short-Term (up to 3 Months) Post-Flood Vulnerability Factors

#### 3.6.1. Mortality

No included studies identify risk factors for short-term mortality, however, the increased proportion of heart attacks observed in two studies may suggest vulnerability among the elderly [48,49]. 

#### 3.6.2. Morbidity

Four studies examined risk factors for gastrointestinal illness (GI) up to three months post-flooding [21,24,50,51]. In the US there was evidence that those in poor to good health (compared to those with excellent or very good health), and those with existing chronic gastrointestinal symptoms (such as irritable bowel syndrome) were at increased risk of GI (self-reported increased rates of vomiting, liquid or soft diarrhea, or nausea with cramps during a single 24-hour period) [21]. In this study, there was no evidence of increased rates of GI among those who consumed public water supply. Among those five years or younger, any flood contact was associated with GI rates (incidence rate ratios (IRR) 3.18, 95 percent CI: 1.79, 5.66). Among those 12 years or younger, the presence of a septic tank on the home property was not associated with increased rates of GI, and although any contact with floodwater or flood-contaminated items was associated with elevated rates of GI, this association was significant only for those who participated in flood clean-up (IRR 1.40, 95% CI: 1.07, 1.82) or whose house or yard was flooded (IRR 2.42, 95% CI: 1.22, 4.82). Additionally, children whose home or property was flooded were at 1.9 times greater risk of increased rates of GI than children whose homes or yards were not flooded [21]. For those over 50 years, flooding of homes or yards was also associated with higher incidence rates of GI (IRR 6.20, 95% CI: 3.34, 11.51). 

A large number of *E. coli* cases (1,346) were identified following flooding of groundwater into municipal water supply [51]. The cross-sectional study showed a dose-response relationship, with occupants of homes connected to and consuming the public water supply 11.7 times more likely to develop GI than those not exposed to public water supply [51]. Just over half (57%) of the cases were female and the median age of cases was 29 years (range < 1 to 97 years), but this was not compared to controls. This study also found residents continued to brush teeth with, and drink water, despite extensive cautionary publicity and a boil water advisory [51]. In a case series (Czech Republic), risk factors for identification of Tahyna virus (TAHV) antibodies were decreasing distance from flood and increasing age, with no differences based on gender [24]. All cases of leptospirosis, identified in an Australian study, were ill within 2 to 30 days from exposure to floods; all were hospitalised, all were male and the median age was 34. All had direct exposure with floodwater and most had cuts or abrasions [50]. 

### 3.7. Health Impacts Observed Long-Term (3 Months Plus) Post-Flood

Two studies examined occurrences of mortality occurring three months or more post-flood [38,39]. One study observed a 50% increase in all-cause mortality among the flooded population in England and an increase in deaths from cancer [38] (see Figure 6). However, a study of 319 flood events in England and Wales identified a reduction in mortality post-flood [39]. The authors concluded that these counter-intuitive results may be biased by displacement of flood-affected individuals (particularly frail and elderly at increased risk of dying) to non-flooded areas (and are therefore not represented in the study) or that the increased support from networks positively affected well-being and reduced mortality [39]. 

Two studies examined rates of mortality attributed to suicides before and three months plus, post-flooding [45,47]. An Australian study found no significant difference in the rates of suicides after severe flooding [45]. The authors did note that the follow up time was short, and often there is a period of support following floods that can act to protect individuals from feeling suicidal, and suggested a follow-up time of two years [45]. Furthermore, while floods were explicitly considered a contributing factor for a single suicide, the authors note that this may be an under-estimate as natural disaster exposure is not routinely collected on the generic form for reporting suicides [45]. The second study also did not find a significant difference in suicide rates after natural disasters (including floods) in America between 1982 and 1989 [47]. 

Six studies examined longer-term post-flooding morbidity [22,23,29,37,38,42] and two studies examined health service-use as proxy for morbidity [25,38]. A range of flood-attributed morbidities were identified during the longer-term timeframe (see Figure 6); including earache and infections [23,37]; post-flood cleanup injuries [22,28]; allergies [28]; throat nose, eye, or skin irritations [22,23,28]; kidney [23] or respiratory conditions [22,23,28]; headaches [23,28]; gastrointestinal illnesses [22,23] and heart and high blood pressure issues [22,23]. Among residents whose homes were extensively flooded in England and Australia, there was an increase in likelihood of being admitted to hospital and in general practitioner (GP) attendance rates [25,38]. A number of studies identified mental health or psychological symptoms post-flood. These included: post-traumatic stress disorder, anxiety when it rains, panic attacks, stress and sleeping problems [22,23,26,27,28,30,31,32,33,38,40,43,44,46]. The effects of flooding on psychological symptoms appear to be long lasting [26,46]. In some studies, it is unclear how much of this morbidity can be directly attributed to being flooded [22].

### 3.8. Long-Term (3 Months Plus) Post-Flood Vulnerability Factors

#### 3.8.1. Mortality

Although two studies examined occurrences of long-term mortality, up to 12 months post-flood, neither study examined the risk factors for mortality among those who were flooded [38,39]. One study, in England, observed an increase in all-cause mortality, particularly among those 45 to 64 years, for males and with a somewhat unexpected rise in deaths from cancer. There was also a significant increase in deaths for adults over 65, especially females over 75 [38], when compared to non-flooded populations. Another study of flooding events in England and Wales identified a reduction in deaths post-flood that did not vary substantially by age, sex, population density or income [39]. 

An Australian study found having had previous suicide attempts and communicating suicidal intent were significant risk factors for those who committed suicide longer-term post-flood [45]. There were no differences in rates of suicide by gender, marital status, ethnicity, employment status, living arrangements, or stressful life event [45]. 

#### 3.8.2. Morbidity

Two studies examined service use as a proxy for morbidity and found hospitalisations (or referrals to hospitals) doubled for males only [25,38]. There was a significant increase in general practitioner attendances of 53% (males 81%, females 25%) and significant increases in multiple attendances for males only. A significant increase in newly reported symptoms was also observed in males (33% flooded males reported new physical symptoms compared with 16% of non-flooded males) in England [38]. For those 1 to 4 years and individuals over 55 years, there was also an increase in attendance rates, with no differences associated with social class. A survey of the health of members of flooded and non-flooded households living in the same suburbs of Brisbane before and after flooding found higher percentages claimed worsened health in the year following the flood, with the exception of those over 75 years who were the least affected group [42]. The lack of a difference between those over 75 whose household were flooded compared to those who were not, may indicate that there is no morbidity effect of floods among that age group or that morbidity also increased in those over 75 years living in flood-affected suburbs even though their households were not flooded [42]. The greatest impact of the floods on health was seen in those over 35 years, who are more likely to be homeowners [42]. The number of GP visits did not differ one year post-flood compared to before [42]. 

The extent of flood exposure appears to be related to morbidity outcomes [25,37]. Among residents whose homes were extensively flooded, there was an increase in likelihood of being admitted to hospital in Australia [25]. Similarly a cohort study in England found, earache and gastroenteritis were associated with greater depth of flooding, as measured by the maximum depth of water in feet over the floor of the lowest habitable room [37]. However the effect of depth of exposure on health may be condition specific, as this study also found no effect of depth of flood exposure on other symptoms, and a counterintuitive trend where greater depth of flooding was correlated with reduced risk of worsening asthma [37]. 

In a US-based survey, respondent characteristics associated with negative physical health outcomes post-flood included adverse event experiences, older age, lower socioeconomic status (defined as financial difficulties), Hispanic ethnicity, and non-US citizenship, while access to healthcare and lack of local (English) language proficiency were associated with reduced risk [28]. In the same study, adverse event experiences, older age, lower socioeconomic status and more serious home damage were significant risk factors for clean-up injuries. In a similar study, among the 41% of Hispanic respondents with one or more respiratory illnesses post-flood significant associations were observed for respondents lacking US citizenship, with lower income, greater local-language (English) proficiency, those exposed to mold, or increased family conflict. Gender and age were not statistically significant factors influencing post-flood respiratory illnesses among these respondents [29].

### 3.9. Psychological Health

Ten studies examined a range of factors that appear to be related to the extent of psychological symptoms associated with flood exposure [22,26,27,28,30,32,38,40,42,46]; the results are presented in the following paragraphs. A number of these were methodologically strong prospective cohort studies, examining risk factors for psychological health post-flood among those flooded [30,31,32,33,37,44].

#### 3.9.1. Degree of Flood Impact

It is not surprising that greater exposure to the impact of floods is related to a greater risk of mental health issues post-flooding. Post-flood depression was related to a greater extent of flood impact [32] and having adverse flood event experiences [28] in the US, or flood trauma experiences [27] in Poland. Among those flooded in England and Wales, risk factors for post-traumatic stress (PTS) were deeper water; contamination by floodwater; less warning time; evacuation; and longer recovery [22]. The study also identified risk factors for worse psychological health as measured by the general health questionnaire (GHQ12 score of 4 or more) among those flooded, including evacuation, contamination by floodwater, and less warning time. Similarly, a 2007 study from the UK found that factors related to the impact of floods on psychological health included disruption to essential services, concern that the floods would affect people’s health, perception of an adverse impact on finances and evacuation [40]. 

#### 3.9.2. Gender and Age

Gender and age appear to influence the risk of longer-term mental health symptoms as a result of flood exposure, but results are inconsistent. In England and Wales, females and all age groups except those over 60 years who were flooded had significantly worse general health than individuals who lived in the same area but were not flooded [22]. The same study suggested that among the flooded, risk factors for PTS were being female and younger than 65 [22]. Two studies support older age as a risk factor for increased mental health effects of floods in Poland [27] and US [28], and adults were at greater risk of distress (scored more than 4 on GHQ12) than subjects of other ages in England [37], yet others [30,32] suggest younger age is a risk factor. A study in Poland reported that females have significantly more psychological symptoms than males [27], while another suggests males are at greater risk [31]. 

There is possibly an interaction between age and gender in the psychological impact of floods. One study found flood-affected females younger than 65 years had more psychiatric symptoms than flood affected males. This gender difference disappeared in those over 65 [42]. Similarly, a study in England observed that flood-impacted females reported significantly higher psychiatric symptoms compared to non-flood-impacted females [38]. 

#### 3.9.3. Location and Residential Status

Where a flooded individual lives is important, at least in some situations. Risk factors for long-term PTS in England and Wales were having problems with insurer, being uninsured, and vulnerable housing (such as a ground floor flat, bungalow or caravan) [22]. Additional risk factors for poor psychological health (GHQ12 scores), were longer time to recovery, and living in rental property [22]. In a US study, for a given rating of flood impact, being residents of small towns and rural non-farming communities had a higher negative impact on psychological health [32]. Although flood impact levels were significantly higher among farm residents, it was small communities without farms that had the highest rates of depression [32]. 

#### 3.9.4. Education and Socio-Economic Status

Several studies identified education and socio-economic status as related to the impact of floods on psychological health. Some US studies found flood-related trauma, distress or depression risk factors include lower income levels [32], occupational status [31] or socio-economic status [28] and in Poland, less education [27]. However, other studies found that deterioration in psychological health post-flood was associated with higher educational level or income (Korea) [30], or that new symptoms of depression were not related to level of education (Puerto Rico) [33]. Finally, a Mexican study found evidence of a strong relationship between education and PTS, but the direction of the relationship was not stated [46]. Comparison of these studies could be confounded by cultural and other factors.

#### 3.9.5. Existing Psychological Factors

There may be psychological factors that determine the impact of flood exposure on an individual’s mental health. A range of psychological traits were found to be either protective or risk factors for PTS [26]. For all measured time points (3 months, 15 months, or 3 years after flooding in Poland), trauma and emotional reactivity were the strongest predictors of intensity of PTS symptoms. There was evidence of an interaction between these two predictors explaining the variance of PTS symptoms 3 years post-flood [26]. Additionally, having had previous psychological symptoms appears to be a risk factor for mental health symptoms following flood exposure in Korea [30], Puerto Rico [33], and the US [32]. Among those flooded, poorer prior health was identified as a risk factor for PTS and worse psychological health, following flooding in England and Wales [22].

#### 3.9.6. Social Connectedness

Floods and other natural disasters are frequently associated with increases in risk factors for suicide. However, a study of 210 undergraduate students who experienced the 2009 Red River Flood in North Dakota, found an association between increased time volunteering in flood efforts and a reduction in the interpersonal risk factors associated with suicide, such as not belonging and feeling burdensome [44]. Similarly, there is evidence of greater impact of floods on post-flood depression in those who are separated or divorced in US [32], yet a study from Korea found a greater impact of floods among those who were married [30].

#### 3.9.7. Other

A range of other factors were identified as risk factors in single studies. For example, being a non-smoker or non-drinker was related to increasing risk of deterioration in health post-flood in Korea [30] as was lack of access to healthcare in US [28]. Interestingly, in the US, being local language (English) deficient or foreign-born appears to be protective against mental health effects of floods [28].

#### 3.9.8. *In Utero* Flood Exposure

Schizophrenia rates of adults, whose mothers were pregnant during the flood disaster of February 1953, were compared with schizophrenia rates of adults who were in utero prior to or after floods using the Dutch Psychiatric Registry [43]. The results suggest no significant association between prenatal exposure to maternal stress and risk of non-affective psychosis as adults in those born to mothers who experienced flooding while pregnant [43].

## 4. Discussion

This paper aimed to identify the individual characteristics, or risk factors, that increase vulnerability to the health effects of precipitation related flood events pre-, during and post- impact in OECD countries.

Pre-flood mortality was observed in one of three studies but there was limited examination of risk factors and no comparison group. Pre-flood morbidity was examined in three studies with no observed pre-flood health events in two [19,48]. However, the third study observed that gastrointestinal illnesses can precede flooding events (coinciding with heavy rainfall) [51]. During-flood-related mortality risk factors include being male, particularly between 10 and 29 years [16], elderly [16,17,35], or intoxicated [16], vehicle use during flood, particularly for reasons other than evacuation [16] and risk taking [16,35,52]. The risk factors identified for during-flood gastrointestinal illnesses were being female, skin contact with floodwater and private water supply [18]. Age above 60 increases the risk of during-flood mental or physical illness [34]. 

Factors increasing vulnerability to injuries post-flood during clean up were being younger than 65 years, having lower socio-economic status, experiencing adverse events from flooding and the extent of flooding to home or property [28]. Risk factors for general physical illness post-flood included age below 45 [30] or above 65 years [28], however one study found no effect of age [31]. Gender was not a risk factor for poor general health in one study [31], while another found being male was [30]. Other risk factors included being a non-smoker or non-drinker, or married [30]; limited access to health care, being a non-US citizen, Hispanic, with greater local language (English) proficiency [28]; medication interruption [41]; experiencing an adverse event from flooding, including greater extent of flooding to home or property [28] and lower socio-economic status (financial difficulties) [28]. The latter was not always the case, as lower socio-economic status or education were found not to be a risk factor for physical health in one study [31] and, in another, lower socio-economic status was protective [30]. Flooded males were more likely than females to increase health care utilization post-flood [25,38]. However, it was unclear if this increase in use was matched by an increase in health symptoms. Perhaps males are more open to accessing health services after floods, than prior to floods, or are more likely to do so after floods, as the clean-up stage may require a medical certificate to secure time off work.

There is evidence floods adversely affect post-flood mental health [22,26,27,28,30,31,32,33,38,40,43,44,46]. The degree of post-flood impact on psychological health was associated with depth of flood, adverse event experiences, lack of warning time, evacuation, and disruption to services [22,27,28,32,40]. Gender (predominately being female [22,27]) and age (younger than 65 [30,32]) appear to influence the risk of mental health symptoms as a result of flood exposure, but these results are inconsistent, with other studies suggesting older age is a risk factor [27,28], while another suggests males are at greater risk [31]. Risk factors for long-term PTS among flooded populations were problems with insurer, prolonged recovery, vulnerable housing [22], and being residents of small towns and rural non-farming communities [32]. There is possibly an interaction between age and gender on the psychological impact of floods, with females and older individuals having greater exposure to flood-related damage and its psychological effects [38,42]. As the studies were not prospective, it is unclear if some of these psychiatric symptoms may have been present before the floods [38], or if health care professionals are more likely to diagnose psychiatric symptoms in females than males. It has been suggested that younger, working males may not be as confronted with home flood-related damage, while females and older individuals are [42]. This lack of exposure to flood-related damage may explain the lack of psychological effects among males under 65 [42]. 

A number of studies identified lower education and socio-economic status as related to the longer-term impact of floods on psychological health [27,28,31,32]. However, other studies found that deterioration in mental health, post-flood, is related to higher educational level or income [30], or not related to level of education [33]. A range of psychological traits were protective or risk factors for PTS [26]. Having had previous psychological symptoms appears to be a risk factor, for mental health symptoms, following flood exposure [22,30,32,33]. There is evidence of greater impact of floods on post-flood depression in those who were separated or divorced in the US [32], yet a study from Korea found a greater impact of floods among those who were married [30]. The presence of psychological effects of floods, beyond 18 months to 3 years, suggests that the impact can be long lasting and these populations may be inadequately supported.

Gastrointestinal illnesses and infectious diseases are common post-flooding [21,22,23,24,50,51,53]. There is inconsistent evidence if increasing age or gender are risk factors for post-flooding gastrointestinal illness [24,50,51]. The effect of the gastrointestinal illnesses was more severe among those in poor, fair, or good health (compared to those with excellent or very good health); and in those with existing chronic, gastrointestinal symptoms (such as irritable bowel syndrome), particularly for severe diarrhea illness [21]. Exposure to floodwater and cuts or abrasions appeared to increase the risk of GI symptoms [50]. One study found that those in homes connected to and consuming the public water supply (and increasing consumption of this water) were more likely to develop gastroenteritis in Canada [51]. Another found no evidence of this, and instead found that contact with floodwater or having flooded homes or yards were risk factors [21]. 

Research suggests that shigella, cholera, norovirus, and dengue, among water or vector borne diseases, are commonly associated with flooding and may need to be monitored during and after floods [54,55,56,57,58,59,60]. Of all extreme weather events, heavy rainfall, with or without flooding, is commonly implicated in waterborne disease outbreaks [53,61], particularly in conjunction with warmer temperatures [62,63]. This suggests the need for increased awareness, surveillance and identification of risk factors associated with vulnerability to water or vector-borne diseases, not just immediately following flood events, but also during heavy rainfalls, particularly following dry spells and prior to floods. The observation that gastrointestinal illnesses can precede flooding events [51] is of interest. This observation is supported by a systematic review [53] and recent finding that rainfall upstream of a public water supply was associated with an increase in calls to a nurse phone line for advice on acute gastrointestinal illnesses [64]. Similarly, a significant increase in cases of cholera and other gastrointestinal illnesses pre-flood has been observed in developing countries [65,66]. The presumed cause is contamination from runoff, preceding a flood, resulting from heavy rainfall [65]. During periods of heavy rainfall, proactive measures to assist in ensuring the continued safety of drinking water supplies should be considered. Proactive measures include strategic water supply system planning to aid infrastructure resilience; identification of standby water sources; early detection of deterioration in quality associated with water surges; and flood mitigation measures [67]. 

The current research compliments earlier research that focused on flood-related exposure susceptibility, recovery, mitigation, preparedness and risk perception, by seeking to identify risk factors associated with the health effects of floods beyond being exposed to floodwater, as not all individuals exposed to floodwater experience health effects. A recent comprehensive review of the human impact of floods overlaps in context and content, however it is focused primarily on flood, population or regional characteristics and their association with human impacts [68]. Another related review examines the health effects that can be attributed to floods, again covering a broad range of flood types, worldwide [69]. The WHO Regional Office for Europe and the Health Protection Agency surveyed a number of European countries to identify mitigation, preparedness and response strategies before, during and after flooding, and additionally examine the health effects of flooding [70]. We focused on precipitation-related floods and OECD countries, as it is likely that there are similarities between these countries in terms of characteristics of extreme precipitation-related flood events, flood mitigation strategies and, risk factors within populations that increase health risks associated with flood events. A limitation of this review is despite the similarities shared by the OECD member counties, comparison of these studies could be confounded by cultural, geographical, political and other factors, as well as within study design differences. 

The vast majority of previous research focuses on determining the geographic or socio-demographic factors that increase vulnerability of household exposure to, or economic impact of, floods and subsequent recovery [11,12,13,71,72,73,74,75,76,77,78]. This focus on risk factors for flood exposure and economic impact is perhaps explained by floods being one of the most costly forms of natural disaster. Vulnerability to exposure is determined in relation to social class, ethnicity, age, risk perception [10,14,78] or other factors [11,12,13,71,72,73,74,75,76,77,78]. In addition, strategies to address the impact of risk perception and individual precautionary behavior, such as personally investing in insurance or sandbags [79], has been extensively investigated [80]. More recently, a novel study used a sociopsychometric framework tool to examine risk perception at the municipality level and its impact on risk reducing behaviour [81]. The study identified associations between reliance on traditional flood prevention and Federal-level flood risk management strategies and preparedness to face future floods, without additional measures, despite previous experience of higher than expected damage from past major flooding events in their municipality. The authors conclude that this optimistic perception of security among those surveyed conflicts with their also high ratings of worry and requests for additional protective measures. Respondents value traditional measures, including dikes and precautionary measures, such as urban flood management, legal restrictions on land use, hazard and risk mapping over risk reduction strategies aimed at individual support (such as public awareness programs, private insurance and financial support).

Studying the risk factors increasing the health effects of floods poses strong methodological issues. The majority of studies were based on case series reports. Case series reports typically report on the proportions of individuals who were flood-health affected and provide crude demographic factors (such as distributions of gender, age, and ethnicity). However, as there is relatively little information on unaffected individuals, or baseline population demographics in research conducted in OECD countries, the factors associated with increased risk cannot be accurately determined. 

Case-control studies can estimate the risk associated with various exposures. There are four case studies of individuals, who experienced health effects during-flood events using controls who were also flooded [19,20,39,43]. Interestingly these studies were typically unable to identify demographic or socio-economic factors that increased vulnerability to health effects of flood events. Although case-control studies provide stronger evidence, there is the potential for recall bias for the factors and exposures assessed. Further, additional problems occur when assessing the risk factors for flood-related mortality, as the researcher’s ability to measure all but the crudest potential exposures is highly limited or requires a proxy to report for the deceased individual. 

A stronger design would be to conduct a prospective cohort study, where a population in a risk area is recruited and various potential risk factors are studied and then followed through a flood event and outcomes measured. Difficulties with this design are that flood events are not predictable, particularly catastrophic ones, individuals will move in and out of flood-prone areas and, for rare events, including mortality, very large sample sizes would be required to identify associated risk factors. Promisingly, seven included studies are cohort studies. One focused on gastrointestinal illnesses [21] and the remainder examined risk factors for psychological health post-flood [30,31,32,33,37,44]. However, we were unable to identify any studies that examined risk factors for mortality among those flooded, pre and post-flood impact. This is a potential area for future research. 

The presence of morbidity and mortality effects immediately prior to flooding (due to evacuation, gastrointestinal illnesses and stress) warrants further investigation to identify potential risk factors. Future research should also separate the risk factors for particular health effects associated with flooding, as the risk factors for gastrointestinal illnesses may well be quite different from psychological effects or respiratory illness. In addition, this research area would benefit from prospectively capturing a wider range of data at baseline (beyond gender and age). This would allow detailed examination of potential risk factors.

This review has potential implications for tailoring of flood-related health messages and programs. Pre-flooding morbidity and mortality risk factors suggest that populations at risk of flooding need reminders that using vehicles, for any other reason than evacuation, increases risk of drowning, and that males, particularly those over 60 years, are at greater risk of heart attacks associated with evacuation and mitigation efforts, such as sandbag preparation. During flooding, providing access to appropriate protective clothing might be useful given the increased risks associated with skin contact with water and cleaning up contaminated property. Post flooding, access to routine medical care (including medications) appears to be important, particularly for those with physical and psychological symptoms prior to flooding. Potentially, individuals with existing conditions should be advised to take precautionary actions to prevent worsening of symptoms, particularly in the context of forecasted wet periods, such as storing sufficient essential medication for the management of their condition (ideally in an elevated location) to ensure continuous coverage. Other pre-flood preventative actions could include seeking mental health support and precautionary boiling water for individuals with chronic gastrointestinal symptoms or a predisposition to such symptoms. 

## 5. Conclusions

The psychological and physiological health effects of floods appear disproportionately borne by females, elderly and children during floods, while males between the ages of 10 and 29 are at greater risk of mortality. Factors increasing post-flood impact include being older than 65 years, males at risk of physical health effects, and females at risk of psychological health effects. However, the literature base is relatively small. Studying the effects of floods is not a trivial undertaking and further research is required to identify the risk factors associated with pre-flood mortality and morbidity and post-flood mortality. Ideally prospective cohort studies should be initiated. Public health messages should target females and young people who appear to be at greater risk of psychological and physical health effects of floods, and males at greater risk of mortality, primarily due to risk taking behavior. Public health messages targeting those at risk post-flooding should include: consideration of those with previous experience of flood, low education or socio-economic status, taking medicines (to avoid medicine interruption) and those with existing psychological, chronic gastrointestinal or cardiovascular illnesses (to avoid worsening symptoms). Future research is required to identify how best to reach these vulnerable population groups.

## Appendix

**Table A1:** Studies addressing research Question 1. What are the demographic or other factors associated with increased risk of morbidity or mortality, among those already flooded?

Reference Country	Study type Event Area of focus	Methods	Findings
Schnitzler, Benzler *et al.* 2007 [18] Germany	Qualitative survey Saxony flood 2002 Risk factors for health (diarrhea or injury)	Random survey of 477 flooded in 42 Saxony communities. Included an analysis of onset of diarrhea or injury during or immediately after flood. Univariate and multivariate analysis of exposures associated with onset of diarrhea or injury. Risk factors analysed were age (51+), gender (female), skin contact with floodwater, indoor living area flooded, cleanup involvement, consuming flood exposed food, drinking private water supply, consuming bottle water, mains water boiled/unboiled, water from tank).	**During-flood:** Thirty-two out of 465 (6.9%) had diarrhea during or shortly after the flood; 55 out of 472 (11.7%), had injuries. Multivariate analysis suggests exposures associated with onset of diarrhea were skin contact with floodwater (OR 5.8, 95%CI: 1.3, 25.1), being female (OR 3.9, 95%CI: 1.5, 10.0) and private water supply (OR 3.5, 95%CI: 1.2, 10.5), flooding indoors was significant for univariate only. A multivariate analysis showed that the risk for injuries was only significantly increased for skin contact with floodwater (OR 17.8, 95% CI: 2.4, 130.5), cleanup involvement and flooding indoors were significant for univariate only. Age was not a risk factor.
Steinführer & Kuhlicke [34] Germany	Qualitative survey Mulde catchment flood August 2002 Risk factors for psychological and physical health	Survey of 404 households affected by the 2002 Mulde catchment flood carried out in five locations. Included a question on health effects both psychological and physical (not otherwise described). Collected social and demographic as well as flood-related risk factors.	**During-floods:** Among those flooded, those over 60 were at increased risk of evaluating both the psychological and physical consequences of floods significantly more often as “very bad”. The very old and very young varied in their perceptions significantly for psychological consequences; p < 0.05). Tenure, gender, and location were not significant predictors of psychological or physical consequences.
Staes, Orengo *et al.* 1994 [20] Puerto Rico	Case-control study Puerto Rico floods 1992 Flood mortality	Descriptive study: time, place, and circumstances of death compared with water-level, rainfall and the timing of official warnings. Case control study: controls selected from the exposed population to estimate the risk of death by age, gender and vehicle occupancy during flood.	**During-floods:** Fatalities: 23; 20 were vehicle occupants and many died prior to official warning as water rose rapidly. Case control results: Gender or age did not alter the estimated mortality risk. Vehicle occupancy elevated mortality significantly [OR 15.9 (95% CI: 3.5, 144)] N.B control group had significantly less income than census details of exposed population and may not be representative exposed population ownership of cars. Vehicle occupancy to evacuate flash flood area was protective; other reasons for vehicle occupancy increased the risk of mortality. Blood alcohol content was positive for 12/16 adult deaths: 5 exceeded 0.1%.
Wade, Sandhu *et al.* 2004 [21] US	Prospective longitudinal cohort Severe flooding in the midwestern United States April and May of 2001 Examined rates of gastrointestinal illness during the flood, stratified for sensitive groups and whether contact with floodwater was associated with increased risk of gastrointestinal illness	Randomized trial of in-home drinking water treatment (the Water Evaluation Trial or “WET” Study) underway at the time of the flooding. Participants completed daily diaries detailing their incidence of gastrointestinal symptoms. 456 households (1,296 persons) were enrolled, and follow-up was for 1 year. A total of 1,110 of 1,118 subjects in the WET cohort who completed the flood survey provided health data, 143 (13%) reported some type of direct (e.g., walking through floodwater) or indirect (e.g., clean up floodwater contaminated items) contact with floodwater. Data was stratified in the models by age (≤12 years and ≥50 years), frequency of gastrointestinal symptoms in past year, and the presence of an existing chronic gastrointestinal condition to examine whether the impact of the flood was greater in certain potentially sensitive groups.	**During-flood:** Crude rates, of both gastrointestinal symptoms and diarrhea, were higher during the flood, than the rates for winter. Rates of gastrointestinal symptoms and diarrhea among, participants were higher in winter compared to other seasons. Adjusted rates of highly credible gastrointestinal symptom episodes were 1.29 times higher during the flood, than during the rest of the follow-up period (95 % CI: 1.06, 1.58). **Post-flood:** Numbers of diarrhea episodes, as well as hospitalizations, for gastrointestinal conditions were non-significantly elevated. Doctor’s visit for diarrhea; days of missed work or school due to gastrointestinal symptoms; and days of vomiting were all non-significant. Six participants were hospitalized for a total of 29 days for gastrointestinal conditions. Among those ≤12 years, the presence of a septic tank on the home property was not associated with elevated incidence rate ratios (IRRs) of gastrointestinal symptoms, and although any type contact with floodwater or flood-contaminated items was associated with elevated IRR of gastrointestinal symptoms, this association was significant only for those who participated in flood cleanup (IRR 1.40, 95% CI: 1.07, 1.82) or whose house or yard was flooded (IRR 2.42, 95% CI: 1.22, 4.82). For the ≥50 years old, any flood contact had higher symptoms (IRR 1.46, 95%CI: 0.65, 3.27) and as did those whose homes or yards had been flooded (IRR 6.20, 95%CI: 3.34, 11.51). Among those ≤5 years any flood contact was associated with credible gastrointestinal symptoms (IRR 3.18, 95%CI: 1.79, 5.66). The effect of the flood was more severe among persons self-rating health as poor, fair, or good; and those with frequent gastrointestinal symptoms (such as irritable bowel syndrome), particularly for severe gastro illness. There was no evidence that gastrointestinal symptoms increased in those who consumed public water nor was there evidence of a dose response relationship.
Tunstall, Tapsell *et al.* 2006 [22] England and Wales	Qualitative study England and Wales Floods 30 locations from 1998 to 2002 Health and psychological effects of floods and the gender, age, socio-economic predictors	Surveys conducted on flooded sample (983 adults 18+ years whose homes had been flooded above floor level) compared with at risk sample (527 residents 18+ in the same areas but who did not experience flooding) general health questionnaire (GHQ-12); post-traumatic stress scale (PTS); self-reported health effects checklist.	**Post-flood:** Suggests that among the flooded risk factors for worse psychological health (GHQ12 scores) were female gender; poorer prior health; problems with insurers/being uninsured; evacuation; longer time to recovery; contamination by floodwater; living in rental property and less warning time. Among the flooded risk factors for PTS were female gender; poorer prior health; being younger than 65; problems with insurer; deeper water; vulnerable housing; contamination by floodwater; less warning time; evacuation; and longer recovery.
Duclos, Vidonne *et al.* 1991 [19] France	Case study (included an injured uninjured case control comparison) Nîmes flood 1988 Flood-health effect (mortality, injury and disease). Age only	Assessed overall flood-health impact by data on medical care delivery & surveillance of infectious diseases. Survey of 108 families (228 persons). Describes 1) the factors that limited mortality, 2) the reaction of the population to the disaster, & 3) the health effects during the impact & post-impact phases of the disaster.	**During-flood:** Among flooded respondents, average age similar for those injured 46.4 years compared with 48 years for all respondents.
Tapsell, Penning-Rowsell *et al.* 2002 [23] England	Qualitative Northeast England floods June 2000 Health effects of flooding & vulnerability mapping	Focus groups 3 to 4 months after floods to determine the health effects of the flood. Developed an index to measure the impact floods may have on communities using SFVI (a composite additive index based on 3 social indicators: age, lone parents, & pre-existing health problems & 4 financial indicators, non-home owners, unemployed, non-car owners, and overcrowding).	**Post-flood:** Reported health problems: blood disorder; chest infections /asthma /coughs /colds /flu /pleurisy; kidney infection; diarrhea/ vomiting/ upset stomachs; headaches; high blood pressure; skin irritations/rashes/ spots; panic attacks; swollen glands; throat and ear infections /laryngitis; viral infections Using SFV1, populations can be categorized into bands of: 1) low 3) average & 5) high vulnerability. Maidenhead flood plains are populated by relatively affluent communities with slightly lower average SFVI values than in the surrounding areas. Manchester flood plains are much more vulnerable to flooding than Maidenhead, with a large, vulnerable community in the southwest of the flood-plain area. More research is needed to determine the accuracy of SFVI scores at predicting actual vulnerability to flooding.
Hubalek, Zeman *et al.* 2005 [24] Czech Republic	Case series Czech floods 2002 Screened the human population of the flooded area to estimate the risk for infections with mosquito-borne viruses	Specimens from residents (N = 497) of an area in the Czech Republic affected by the 2002 flood were examined serologically for mosquito-borne Tahyna (TAHV), Sindbis (SINV), Batai (BATV) viruses, and West Nile (WNV) viruses. Determined the difference in rates based on 4 zones, proximity to flooded areas, gender and age.	**Post-flood:** Antibodies were detected against Tahyna (TAHV) (16%), Sindbis (SINV) (1%), and Batai (BATV) (0.2%) viruses, but not West Nile (WNV) viruses. Association found with decreasing distance from floodplain and increasing prevalence of TAHV seroconversion (χ2 = 8.51; p = 0.003) for Zones A, B and C. The highest TAHV seroprevalence in zone A (28%), lower seroprevalences in zones B and C, and 5% in the control zone D (χ2 = 14.57; p = 0.002). There were no differences in TAHV seroprevalence based on gender, (15.8%) males and (16.9%) females (χ2 = 0.11; p = 0.744). The prevalence of TAHV increased significantly with increasing age (χ2 = 39.809; p <0.001). Four cases of TAHV were observed in testing, but not corroborated by GP (suggesting mild symptoms). No recent cases of infection to WNV, SINV, and BATV viruses were observed in study.
Handmer & Smith 1983 [25] Australia	Comparison Flooding in Lismore Australia, 1974 Hospital admission and mortality risks associated with flooding	Used data from hospital admission and death certificates and from an earlier survey. Compared mortality and hospital admissions before and after the flood; and differential health effects by level of flood and gender; included residents outside flood plain.	**Post-flood:** there were gender differences among the severely flooded, admissions doubled for males, while female admissions halved.
Strelau, Zawadzki *et al.* 2005 [26] Poland	Cross-sectional Great Polish Flood (Southern Poland) in 1997; Northern Poland floods in 2001 Post-traumatic stress (PTS) disorder predictors	Four studies of flood victims (562/1041 were female). We focus here only on those flooded (study included other disaster events). Post-traumatic stress disorder symptoms (PTS-Factorial Version inventory) were measured at varying time points (3, 15 months, or 3 years after flooding). Slight differences in methods between studies, however common measures included Trauma Intensity index, which examined threat to life during the flood, injuries of the body, and material damage. Prolonged trauma consequences index including financial problems; problems with housing; and decline in SES after the flood. Temperament Inventory comprised six scales: Briskness, Perseveration, Sensory Sensitivity, Endurance, Emotional Reactivity and Activity.	**Post-flood:** Among those who experienced floods, PTS scores decreased between a few weeks to two years after trauma. Of the traits, emotional reactivity and perseveration positively correlated with intensity of PTS symptoms however, briskness, endurance, and activity were negatively correlated. Sensory sensitivity did not count, when the first measure of PTS was taken into account, but at two years after flood this temperament trait correlated negatively with PTS. For all time points (3 months, 15 months, or 3 years after flooding) trauma and emotional reactivity were the strongest predictors of intensity of PTS symptoms experienced during the flood. Predictors PTS intensity symptoms at 3 years post-flood were emotional reactivity and prolonged trauma consequences of flooding and there was evidence of an interaction between the two predictors explaining, the variance of PTS symptoms.
Norris, Kaniasty *et al.* 2002 [27] Poland	Cross-sectional Flooding in Poland 1997 Post-traumatic stress disorder (PTS using a 30-item Revised Civilian Mississippi Scale) and effect of age	Purposeful sample of flood- affected. Symptoms of post-traumatic stress disorder (PTS) were measured (6–12 months) post-flood (n = 285). NB study also looked at impact of hurricanes in US and Mexico but this is beyond the scope of this review.	**Post-flood:** Women reported significantly more symptoms than men (t = 5:22, p ≤ 001). Symptoms increased as trauma increased (t = 6:51, p ≤ 001) and increased with decreasing education (t = 3:98, p ≤ 001). Among those flooded, there was a linear and positive relation with PTS with older people being most distressed.
Collins, Jimenez *et al.* 2013 [28] US	Cross-sectional survey (retrospective) Flooding in El Paso County, Texas US, 2006 Physical health, mental health and injury post-flood and logistic regression of independent variables	Surveyed, by mail 475 individuals, whose homes were flood damaged four months following flood event. Ten independent variables including: flood exposure (serious home damage, adverse event experiences), gender, age, socio-economic status, access to medical care, Hispanic ethnicity, US citizenship status, foreign-birth, and English-language proficiency.	**Post-flood:** Survey respondents had high rates of physical (43%) or mental (18%) health problems in the 4 months post-flood and 28% had one or more injury, or acute effect, related to post-flood cleanup. Common physical health problems included allergies, throat irritations/coughing/wheezing, headaches and nose/eye/skin irritations. Mental health problems included depression (17%) and PTS (8.6%). Injuries and acute effects, related to cleanup, were stiffness/ soreness, strained muscles and bruises/ sprains/ abrasions. Negative physical and mental health outcomes post-flood, were associated with adverse event experiences, older age, lower socio-economic status, lack of access to healthcare, non-US citizenship and English proficiency. Hispanic ethnicity was associated with physical health. Native-birth was associated with mental health. Adverse event experiences, older age, lower socioeconomic status and more serious home damage were significant risk factors for clean-up injuries. Flooding resulted in higher negative health effects among people more exposed, poorer, older, and with less resources. Hispanic ethnicity and a lack of US citizenship were associated with higher risks of health effects, being English-deficient appears to be protective against physical and or foreign-born protective for mental health effects of floods.
Jimenez, Collins *et al.* 2013 [29] US	Cross-sectional survey (retrospective) Flooding in El Paso County, Texas US, 2006 Respiratory health and relationship with age, gender, SES, mold exposure, family conflict, English-language proficiency and US citizenship status among those with Hispanic ethnicity	4 years post-flood retrospective mail-out survey assessed respiratory health effects for 363 people (176 households), who self-identify Hispanic ethnicity and whose homes were damaged by flood. Analysis of respiratory health and the relationship with age, gender, SES, mold exposure, family conflict, English-language proficiency and US citizenship status, among those with Hispanic ethnicity, was assessed, using logistic regression.	**Post-flood:** Among Hispanic respondents 41% had one or more post-flood respiratory illnesses. Significant associations with respiratory illness were observed among Hispanic respondents with lower income (OR: 0.53 95%CI: 0.36, 0.78), exposed to mold (OR: 2.27, 95%CI: 1.56, 3.29), or increased family conflict (OR: 1.45, 95%CI: 1.05, 2.01), with greater English-language proficiency (OR: 4.02, 95%CI: 1.91, 8.50) or lacking US citizenship (OR: 13.11, 95%CI: 1.75, 98.33). Gender (female OR: 1.36 (95% CI: 0.75, 2.46) and age (under 15 years OR: 1.30 (95%CI: 0.68, 2.47); over 64 years OR: 0.64 (95%CI: 0.17, 2.38)) were not significant factors for post-flood respiratory illnesses.
Ginexi, Weihs *et al.* 2000 [32] US	Prospective cohort study before and after floods Flood Iowa US 1993 (Midwest floods) Depression (CES-D scale) and socio-demographic modifiers pre and post-flood among those exposed to flood effects and those unexposed to flood impact	2379 people (over 18 years) were randomly sampled and assessed 1 year, pre- flooding. 1735 people were assessed 30 to 90 days post-flooding. 893 respondents were impacted. Risk factors for depression, including age, gender, education, marital status, race and income, and community size, were sought during telephone interviews. Those, who were not followed up, were more likely to be male, never married, with slighter lower SES, depressed pre-flood, and reside in non-farm, rural communities. While the means and variances were affected by attrition, the overall relationship between independent variables and depression were not.	**Post-flood:** significant predictors of post-flood depression included pre-flood depression (OR 8.6, 95%CI: 5.54, 13.21), flood impact level (OR 1.10, 95%CI: 1.02, 1.18), age (OR 0.98, 95%CI: 0.96, 0.99), income (OR 0.84, 95%CI: 0.76, 0.94) and those separated or divorced (relative to those married p˂0.001). Multiplicative interactions were observed for elevated post-flood depressive symptoms among males, those with lower SES, and residents of small towns and rural nonfarm communities. Although flood impact levels were significantly higher among farm residents, it was small communities without farms that had high rates of depression.
Heo, Kim *et al.* 2008 [30] Korea	Prospective before and after study Flood July 15th, 2006, Korea (Garisan-ni, Inje-gun, Gangwon-do) Health (SF-36); depression (Beck Depression Index) post-traumatic stress disorder (Minnesota Multiphasic Personality Inventory and the Revised version of the Korean Impact of Event Scale)	A brief survey of 83 subjects was completed two weeks prior to floods. A follow-up post-flood (18 months) survey sought data from 58 of the original subjects on: general health status, depression, PTS, and potential predictors and confounders of mental health outcomes. Survey included: demographic data, (age, gender, and marital status) of the respondents.	**Post-flood:** 6 of the original subject, died due to flood. At follow-up, 53% respondents were, at least, mildly depressed and 17% had severe depression, 22.41% had PTS (as measured by both the IES-R and MMPI-PTS). Of the eight SF-36-K health status categories, physical functioning, role limitation due to emotional conditions, social functioning, and bodily pain were impaired post-flood. General health, role limitation due to physical conditions and vitality were improved post-flood. Logistic regression of the 64% who had deteriorated health post-flood (a reduction of 1 or more in SF-36K), suggests factors associated with this reduction included previous experience of a number of disaster events and those with a score indicating more than mild depression on the BDI. Demographic characteristics increasing risk of deterioration in health post-flood included being a non-smoker or non-drinker, younger, male, married, or having higher educational level or income.
Phifer 1990 [31] US	Prospective before and after cohort study Flooding in southeastern Kentucky US, 1984 Examined effect of age, gender, marital status, occupational status, education level, pre-flood anxiety, depression, well-being and general health before and after flood	200 adults (55 years and older) were interviewed before and after flood to determine differential vulnerability to increases in psychological and physical symptoms by age, gender, marital and occupational status, education level, and pre-flood symptom levels anxiety (State -Trait Anxiety Inventory), depression (Center for Epidemiologic Studies Depression Scale); well-being (General Well-Being Scale) and general health (from a revised 20-item self-report scale of functional health and specific ailments) before and after flood. Follow-up was 18 months.	**Post-flood:** Flood-impacted (*i.e.,* those respondents reporting personal losses) and unexposed groups were similar on distributions of sex, education, occupational status, marital status and pre-disaster symptoms. The only significant difference was in terms of age distribution, where the age 65-74 group was under-represented among the impacted group χ2 (N = 222) = 5.14, p < 0.03). The flood had effects on anxiety, depressive and physical symptoms, when measured at 16-18 months post-flood. Risk factors for psychological symptoms post-flood were being male, lower occupational status and those 55–64 years. Socio-demographic factors do not appear to increase risk, of deterioration of physical health, post-flood.
Canino 1990 [33] Puerto Rico	Prospective cohort study, before and after floods, un-impacted served as controls; combined with retrospective cohort Flooding and landslides Puerto Rico 1985 Mental health (major depressive episode, dysthymia, post- traumatic stress disorder (PTS), alcohol and drug abuse/or dependence (DAD), generalized anxiety (GA), panic, and antisocial personality disorder (ASP)) Diagnostic Interview Schedule/Disaster Supplement (DIS/DS)	912 interviews post-flood (375 were prospective sample and 537 retrospective sample). Note that PTS, GA, DAD, and ASP was not assessed in 1984; so no pre-flood comparison is available for these outcomes. Interviews were conducted in 1887, flood occurred in 1985). 77 of the prospective sample were exposed to the flood (significantly more males exposed than females), half retrospective sample were exposed to the flood. In both samples, the exposed were significantly less educated than the unexposed, but did not differ on other characteristics.	**Post-flood:** New symptoms could not be explained by risk factors, such as, sex, age, education, and previous symptoms.

**Table A2:** Studies addressing research Question 3a: What are the characteristics of individuals who have experienced flood-related morbidity or mortality, with reference to a source population?

Reference Country	Study type Event Area of focus	Methods	Findings
FitzGerald, Du *et al.* 2010 [35] Australia	Historical case series Australian floods (1997–2008) Deaths (demographic only- age and gender)	Flood fatality data in Australia (1997–2008), derived from newspapers & historic accounts, government & scientific data on the date, location, age, gender & cause of death.	**During-flood:** 73 deaths. Gender: males 71.2%. Age: those between 10–29 and 70+ years are over represented among those drowned (not comparative with source populations). Cause: 48.5% fatalities related to motor vehicle use, 26.5% fatalities occurred as a result of inappropriate or high-risk behaviour during floods (*i.e.*, swimming in or trying to surf in flooded water ways); 16% were associated with crossing in flooded water ways.
Thacker, Lee *et al.* 2008 [17] US	Cross-sectional study of deaths Summary of mortality reports from 1979–2004, Overview of deaths from all natural events (report here on flood-related), in United States, demographic vulnerability, ethnicity, gender and age	Using National Center for Health Statistics (NCHS) Compressed Mortality File crude death rates were calculated by dividing the number of condition-specific deaths by the 2000 US census population and converting the rate to per million people. Demographic characteristics of the groups affected are described by age, race, gender, geographic location & year of death.	**During-flood:** 2,741 of the 21,491 (13%) deaths, due to natural events are from storms and floods. Crude death rates did not vary between race and gender. Highest death rates were among those 55+. All age categories had a death rate of less than one per million.
Coates 1999 [36] Australia	Historical case series report Flooding events between 1788 and 1996 Flood deaths	Flood fatalities in Australia compiled from sources; activity of death and death rates in year age intervals, from 0 ± 4 years up to 85 years and older. Population figures were used to calculate a death rate per 100,000 population. The total fatalities, within the population, were divided by the annual, 10, or 50 year average annual population figure for that group.	**During-flood:** From 1788 to September 1996 at least 2,213 flood deaths occurred in Australia. For 1,513 fatalities, gender was reported, 80.6 per cent were male. Increase in fatalities among those 59+ & less than 25 years & slight increase in 35–54 age group. The vast majority of female fatalities were in the 80–84 age group. 1947 to 1996 data show a general increase in male fatalities with age, particularly middle-age males (35–54 years). Most fatalities are from attempting to travel across floodwater (38.5%), being inside a building or campsite (31.5%) or attempting to rescue someone/something else.
Ashley & Ashley 2008 [16] US	Review of case series US all floods from 1959–2005 Mortality by activity; location and demographics	Review of database of 1959–2005 flood-related fatalities compiled from the National Climatic Data Center’s (NCDC) Storm Data. Included data on: flood event type, year, season and state; activity/location surrounding the incident and demographics (age and gender) of a total of 4,586 flood-related fatalities in United States. Study only included those fatalities directly attributed to floodwater (and not those indirect e.g., carbon monoxide poisoning).	**During-flood:** Av. /year 97.6, flood-related fatalities (median value 81/yr). Suggests 10–29 and 60+ years are most vulnerable to flood-related deaths and this is higher statistically relative to the percent of the U.S. population (United States Census Bureau 2000). Those 30–59 years appear less vulnerable to flood-related fatality relative to the percent of the U.S. population. Males comprised the majority of flood fatalities (where gender was known) and among them, 35% were 10–29 years. However, age was not known in 63% of fatalities, while for 49%, gender was unknown. Of flood-deaths, 64% were attributed to an activity or location of occurrence; of these, 63% were vehicle related, 19% in/on permanent structures or outside or alongside the flood (*i.e.*, accidental), 9% were intentionally inside flood-water (of these, 12+ years: 43% walked through floodwaters to evacuate, or reach car/house; 16% entered floodwater to help others).

**Table A3:** Studies that address research Question 2: What are the health effects of floods when compared to un-flooded groups?

Reference Country	Study type Event Area of focus	Methods	Findings
Duclos, Vidonne *et al.* 1991 [19] France	Case study (inc. injured uninjured case control comparison) Nîmes flood 1988 Flood-health impact (mortality, injury and disease) Age only	Assessed overall flood-health impact by data on medical care delivery & surveillance of infectious diseases. Survey of 108 families (228 persons). Describes: (1) factors that limited mortality, (2) reactions of the population to the disaster, (3) health effects during the impact & post-impact phases of the disaster.	**Pre-flood:** No incidence of death **During-flood:** Fatalities: 9 (drowning, 2 rescuers) Injuries: 3 severe (1 burns, 1 fractured leg. 1 broken arm; 2 hypothermia; 3 near drowning; & 10 minor injuries **Post-flood:** No deaths; 12 twelve cases of carbon monoxide poisonings; 3 chemical exposures and few sprains. 2 cases of typhoid fever were confirmed by culture, suspected waterborne, unlinked regionally. Survey results: 32% had flood-related health problems; of these 59 reported stress-related problems (insomnia/ anxiety); other health problems included: influenza, bronchitis, rhinitis, sinusitis & rheumatism. Only 2% with routine scheduled medical treatments or drug prescriptions, experienced flood-related difficulty obtaining medical care.
Reacher, McKenzie *et al.* 2004 [37] England	Cohort study Qualitative Lewes flood 2000 Heath effects of flooding	103 flooded households (227 residents) and 104 non-flooded households (240 residents) in same area randomly selected for the survey. Interviews took place, over the phone, 9 months after flood.	**Post-flood:** Flooding was associated with earache (RR 2.2 [95%CI: 1.1, 4.1]), and a significant increase in risk of gastroenteritis with depth of flooding (RR 1.7 [95%CI: 0.9, 3.0]). For flooded adults risk of worsening asthma (RR 3.1 [95% CI: 1.2, 4.4]) and distress (score more than 4 on GHQ-12) (RR 4.1 [95%CI: 2.6, 6.4]) were higher than non-flooded. Weaker associations were observed for skin rash (RR 3.4 [0.8, 15] p = 0.1), respiratory illness (RR1.3 [0.8, 2.1] p = 0.32) and all categories of injury (RR 1.6 [0.9, 2.8] p = 0.14) (table 2). Sprains, broken bones, burns or scalds, and inhalation of gas, smoke or vapours were reported by flooded and non-flooded individuals. Among the respondents with pre-existing asthma, a non-significant association was observed (RR 1.9 [0.8, 4.2] p = 0.13) for worsening asthma. Associations between flooding and new episodes of physical illness in adults diminished after adjustment for psychological distress. Flooding remained highly significantly associated with psychological distress after adjustment for physical illnesses.
Tunstall, Tapsell *et al.* 2006 [22] England and Wales	Qualitative study England and Wales Floods 30 locations from 1998 to 2002 Health and psychological effects of floods and the gender, age, socio-economic predictors	Surveys conducted on flooded sample (983 adults 18+ years whose homes had been flooded above floor level) compared with at risk sample (527 residents 18+ in the same areas, but who did not experience flooding) general health questionnaire (GHQ-12); post-traumatic stress scale (PTS); self-reported health effects checklist.	**During-flood:** Up to 64% had a score of 4+ on GHQ-12 (psychological distress). **Post-flood:** Psychological effects were much more common after flooding than physical ones, with the most frequently mentioned symptoms being anxiety when it rains; 25% respondents experienced deterioration of health (10% gastrointestinal illness; 9% joint stiffness; 8% respiratory illnesses; 7% high blood pressure and 6% skin conditions) Significant differences between GHQ-12 scores for flooded and those at risk for all age groups except those 60+ years; differences were significant for gender, social class, length of residence (5 years). Gender and age effect was also seen when compared with national average GHQ-12 scores. More than 2/5 flooded perceived the flood as a traumatic event. 15% had mild- moderate PTS; 10 individuals had high and 4 had extreme.
Bennet 1970 [38] England	Controlled survey before and after study Bristol flood 1968 Deaths, hospital referrals and admissions and GP attendance compared with a year prior to floods and following flood. Demographic and social class vulnerability	A comparison was made between people who had been flooded and people who had not, with regard to surgery attendances, hospital referrals and admissions, immediately following the flood, regarding the year before and again the year after. A controlled survey of number of deaths, from flood affected addresses, in the 12 months before and the 12 months after the floods was compared with those from the rest (not flooded) of the city.	**Post-flood:** 50% increase in all-cause mortality among the flooded population in the 12 months following flooding, with a notable rise in deaths from cancer. Highest rise in the age group 45 to 64. Male deaths rose from 7 to 20 and female deaths from 5 to 9 mainly during the third three month period following the flooding. Also significant rise in adults 65+ especially females 75+ (a rise from 9 to 19). GP attendances rose by 53% (males 81%, females 25%), those between 1 and 4 and 55+ years and over had increased attendance rates, but there were no differences associated with social class. Subgroup analysis, of those who were extensively flooded and those who were not re-housed, showed significant shift in attendance pattern (0–2 or 3+ GP attendances) for males (non-significant increase for females). Referrals to hospital and hospital admissions more than doubled, significant in males only. Significant increase in new symptoms in flooded group the year after, 33% flooded males reported new physical symptoms compared with 16% of non-flooded males. Among the flooded females, 18% reported psychiatric symptoms (including psychiatric symptoms which might have been present before the floods), but only 6% of the non-flooded females did so.
Milojevic, Armstrong *et al.* 2011 [39] England and Wales	Case-controlled interrupted time-series analysis 319 Flood events in England and Wales, 1994–2005 Long-term flooding mortality	Compared relative change in mortality, for pre-flood year/ post-flood year deaths in flooded & control (within 5 km of flood) areas. Results were stratified by age group, gender. disease classification (ICD-9, ICD-10), cause of death, urban rural status, quintile of the Index of Multiple Deprivation score for the LLSOA of residence and place of death as on death certificate.	**Post-flood:** 771 deaths, in the year before flooding, and 693 deaths, in the year after (post-/pre-flood ratio of 0.90, 95% CI 0.82, 1.00). This flood ‘deficit’ of deaths did not vary substantially by age, sex, population density or deprivation. Concludes that results are counter-intuitive, may be biased by displacement of flood affected individuals (particularly frail and elderly at increased risk of dying & therefore not represented in the study) to non-flooded areas or that the increased support, from networks, positively effects well-being & reduces mortality.
Paranjothy, Gallacher *et al.* 2011 [40] UK	Qualitative survey 2007 UK floods in South Yorkshire and Worcestershire Prevalence and risk factors for mental health	A population-based survey (n = 2,166) to identify prevalence of, and risk factors for, the psychosocial effects of the 2007 floods in the United Kingdom (3–6 months after floods). Examined psychological distress (GHQ-12), anxiety (GAD-7), depression (PHQ-9), and post-traumatic stress disorder (PTS check list short form) compared to individuals whose homes were not flooded. Also examined risk factors: concern that the floods would affect people’s health; perception of an adverse impact on finances; disruption to essential and evacuation.	**Post-flood:** Prevalence of each mental health measure was significantly higher for those who reported floodwater in the home: psychological distress (GHQ-12) 69%, probable anxiety (GAD-7) 48%, probable depression (PHQ-9) 43%, probable post-traumatic stress disorder (PTS check list short form) 22%, compared to individuals whose homes were not flooded. Risk factors, associated with all mental health measures, were considered in the adjusted analysis and an association was seen for all mental health measures for: concern that the floods would affect people’s health (OR 3.0–4.7); perception of an adverse impact on finances (OR 1.8–3.2); disruption to essential services (OR 1.8–3.1). Evacuation was associated with psychological distress (OR 1.7; 95% CI 1.2, 2.5) only.
Tomio, Sato *et al.* 2010 [41] Japan	Cross-sectional survey Flash flood 2005 Kagoshima, Japan Medication interruption risk factors	Cross-sectional survey of 810 individuals who attended 15 medical facilities.	**Post-flood:** Elderly and chronically ill are at high risk for interruption of medications and those who experienced interruption of medication were more likely to have deteriorated health status one month after the flood (OR 4.5; 95% CI: 1.2, 17.6).
Price 1978 [42] Australia	Case controlled survey and before (immediately following) and 1 year after based study. Brisbane floods 1974 Longer-term vulnerability (demographic: age and gender) to psychological and physical health effects of floods	Survey of the mental and physical health of 246 flooded households (695 people, 69 who were 65+) compared with that of 194 non-flooded households (507 persons, of whom 59 who were 65+) living in the same suburbs of Brisbane. Compared (a) the health of the flooded before the flood with their health afterwards, and (b) the health of the flooded after the flood with that of controls during the same period.	**Post-flood:** Higher proportion claimed worsened health the year following flood, except those 75+ who were the group least affected by the flood experience. The impact of the floods on health increased in 35+ (more likely to be householders). GP visits did not differ in the year after the flood compared to before, however, the young and the very old were likely to have changed their pattern of attendance to GPs after the flood compared to control. Females under 65 years had more psychiatric symptoms than males, but this gender difference disappeared in the 65+ group (working age males not constantly confronted with home damage, like other age groups were).
Selten, van der Graaf *et al.* 1999 [43] Netherlands	Case control Netherlands Flood 1953 Longer-term psychosocial effect of disaster exposure on unborn	Data from the Dutch Psychiatric Registry was examined for an effect of the flood disaster of February 1953. Compared rates of schizophrenia for babies born to mothers who were pregnant during flood and those in utero before or after floods, (but not during).	**Post-flood:** No significant association between prenatal exposure to maternal stress and risk of non-affective psychosis in those, born to mothers, who experienced flooding.
Gordon, Bresin *et al.* 2011 [44] US	Cohort North Dakota 2009 Flood Effect of natural disaster on the desire for suicide	Sample of 210 undergraduate students were surveyed for interpersonal risk factors associated with the desire for suicide (feeling like one does not belong and feeling like one is a burden on others).	**Post-flood:** Association found between greater amounts of time spent volunteering in flood efforts and increased feelings of belongingness and decreased feelings of burdensomeness.
De Leo, San Too, *et al.* 2013 [45] Australia	Case control rate comparison Queensland floods Jan 2011 Suicide rates and characteristics	Examined the rates, and characteristics of suicides, compared to the same time the previous 11 years (based on Australian Bureau of Statistics population numbers for 2000–2010), 6 months after severe flooding in two Queensland towns (Ipswich and Toowoomba). Poisson regression for linear and nonlinear trends in location based suicides; chi-square tests for characteristics of suicide, and Fisher’s exact tests, where counts were less than five in 20% of cells.	**Post-flood:** No significant difference in suicides, compared to the same time the previous year, six months after severe flooding, in two Queensland towns. Follow up may have been too short, and the period of support following floods may have acted to protect individuals from feeling suicidal. Suggest a follow-up time of two years. Previous suicide attempt and communicating suicidal intent were significant risk factors for those who committed suicide post-flood. Among those that committed suicide, there were no differences in rates of suicide by gender, marital status, ethnicity, employment status, living arrangements, or stressful life event, in those that were flooded in 2011 compared to the previous 11 years. For a single suicide, floods were explicitly attributed as one of the contributing factors, however, the authors note that natural disaster exposure is not routinely collected on the generic form for reporting of suicides.
Handmer & Smith 1983 [25] Australia	Comparison Flooding in Lismore Australia, 1974 Hospital admission and mortality risks associated with flooding	Used data from hospital admission and death certificates and from an earlier survey. Compared mortality and hospital admissions before and after the flood; and differential health effects by level of flood and gender; included residents outside flood plain.	**Post-flood:** While there was no overall difference in hospital admissions or deaths pre-flood compared to post-flood, residents whose homes were exposed to a metre or more of floodwater over floor level were twice as likely to be admitted to hospital as residents of the flood free areas.
Norris, Murphy *et al.* 2004 [46] Mexico	Interview and between city comparison Flooding and landslides in Tezuitl’an, Puebla and Villahermosa, Tobasco Mexico, 1999 Post-traumatic stress disorder (PTS) and major depressive disorder (MDD)	561 participants, who were exposed to landslides or floods in Mexico, were interviewed and assessed four times, at 6 month intervals, 6 months post-flood, to examine the course of post-flood PTS symptoms and other outcomes over time. 500 participants, who were located in two flooded towns were interviewed and assessed four times at 6 month intervals (starting 6 months post-flood), to examine the course of post-flood PTS symptoms, and other outcomes over time.	**Post-flood:** PTS was highly prevalent (24% combined sites). Analyses of mean data for counts of PTS symptoms indicated that PTS symptoms initially decreased, but then stabilized around 18-months post-flood. If recovery is not achieved by this time, PTS is likely to be chronic (in approx. 1/3 of cases). For many people recovery occurred after 1 year, suggesting distress may be quite prolonged in the aftermath of floods. Evidence of a strong (F (1, 557) = 51.43, p < 0.001) relationship between education and PTS was observed but the direction of the relationship is unclear from the study report.
Ginexi, Weihs *et al.* 2000 [32] US	Prospective cohort study before and after floods Flood Iowa US 1993 (Midwest floods) Depression (CES-D scale) and socio-demographic modifiers pre and post-flood, among those exposed to flood effects and those unexposed to flood impact	2,379 individuals (18 years or older) were randomly sampled and assessed 1 year, pre- flooding and 1,735 respondents were assessed 30 to 90 days post- flooding. Data on risk factors for depression including age, gender, education, marital status, race and income, and community size were sought, during telephone interviews. Those who were not followed up were more likely to be male, never married, with slighter lower SES, depressed pre-flood, and reside in non-farm, rural communities. While the means and variances were affected by attrition the overall relationship, between independent variables and depression, were not. Impacted respondents numbered 893.	**Post-flood:** Depression scores were, on average, higher among those impacted compared to control respondents, however, the number with depression was not different between groups.
Canino, Bravo *et al.* 1990 [33] Puetro Rico	Prospective cohort study before and after floods; unimpacted served as controls; combined with retrospective cohort Flooding and landslides Puetro Rico 1985 Mental health (major depressive episode, dysthymia, post- traumatic stress disorder (PTS), alcohol and drug abuse/or dependence (DAD), generalized anxiety (GA), panic and antisocial personality disorder (ASP)) Diagnostic Interview Schedule/Disaster Supplement (DIS/DS)	Total 912 interviews post-flood (375 were prospective sample and 537 retrospective sample). Note that PTS, GA, DAD, and ASP was not assessed in 1984; so no pre-flood comparison is available for these outcomes. Interviews were conducted in 1887, flood occured in 1985). 77 of the prospective sample were exposed to the flood (significantly more males exposed than females), half retrospective sample were exposed to the flood. In both samples, the exposed were significantly less educated than the unexposed, but did not differ on other demographic charactersitics.	**Post-flood:** Among the retrospective and prospective samples, there was a trend for the exposed group to have a non-significant higher rate of new cases of depressive disorders and alcohol abuse and/or dependence, than the unexposed. For level of depressive symptoms, in both the retrospective and prospective samples, the differences between groups reached significance. New somatic symptoms and the total number of symptoms in the retrospective sample were found to be significantly more frequent in the flood exposed group. Significant differences for PTS and generalized anxiety, were observed in retrospective sample, in exposed group compared to unexposed, however, these conditions were not measured in the first interview for the prospective sample.
Krug, Kresnow *et al.* 1999 [47] US	Archival case series Floods in America, between 1982 and 1989 Suicide rates	Examined predisaster and postdisaster suicide rates per 100,000 population, 1982 to 1989. Outcomes for earthquakes, hurricanes, severe storms and tornados are beyond the scope of the review.	**Post-flood:** Study found that there was no significant difference between the pre-flood and post-flood suicide rates per 100,000 population.

**Table A4:** Studies addressing research Question 3b: What are the characteristics of individuals who have experienced flood-related morbidity or mortality, without reference to source population?

Reference Country	Study type Event Area of focus	Methods	Findings
Jonkman and Kelman 2005 [48] Europe and US	Case series Worldwide 13 flooding events Deaths and demographic description: age and gender only	247 flood fatalities from 13 flood disaster events, analysed to determine cause and circumstances of death.	**Pre-flood:** 0% deaths **During-flood:** Age at death: <20 yrs = 13.4%; 20–60 yrs = 39%; over 60 = 16.6%, not reported = 30.4%. N.B: Cannot determine age related vulnerability without age distribution of the flood effected population. Gender: assuming that there is an equal gender distribution in the flood affected population males (58.7%) appear to be at great risk of flood mortality than females (25.1%) NB gender was unknown for 16.2%. 75.7 % deaths (83 for Europe) (104 for US), drowning (167) 67.6%; all physical trauma (29) 11.7%; heart attack (14) 5.7%; electrocution (7) 2.8%; carbon monoxide poisoning (2) 0.8%; fire (9) 3.6%; other (3) 1.2%; unknown or not reported 16 (6.5%). Overall: numbers of flood deaths are due to unnecessary risky behaviour. Suggestions of increased vulnerability of the elderly to heart attacks. **Post-flood:** 10.9% of deaths related to clean-up (heart attack and vehicle-related drowning) (4 in Europe and 10 in US)NB 13.4% timing of death not determinable for 8 in Europe and 24 in US.
French, Ing *et al.* 1983 [52] US	Historical summary Various flash floods during 1969–1981 Mortality	A summary of the National Weather Service survey reports on flash floods issued during 1969–1981 to determine the flood mortality, the effect of warnings on mortality, and the cause of death.	**During-flood:** A total of 1,185 deaths were associated with the 32 flash floods, an average of 37 deaths per flood. Of 190 deaths with cause, 93% were due to drowning and 42% of these drownings were car related. The other drownings occurred in homes, at campsites, or when persons were crossing bridges and streams. Other deaths were due to trauma, heart attacks, electrocution or being buried in mud slide.
Jonkman 2005 [1] Worldwide	Database analysis Worldwide flooding events between 1975–2002 (n = 1,902) Loss of life statistics from the OFDA/CRED database concerning a large number of flood events worldwide	Using the Centre for research on the epidemiology of disasters (CRED) & United States Office for foreign disaster assistance (OFDA) databases, analysed flood events between Jan 1975 & June 2002.	**During-flood:** No significant differences between continents for average mortality per flood event (=number of killed/number of affected). Significant regional (17 regions as defined in EM-DAT) differences observed for average flood mortality mainly caused by the dominance of some high mortality events in the regional datasets. No indication of a relationship between mortality and the underlying determinants of the region.
Duclos and Isaacson 1987 [49] US	Case series Floods in Illinois, Oklahoma, Missouri, Michigan 1986 Deaths and demographic vulnerability: gender only	Description of the 24 deaths due to flood.	**Pre-flood:** 3 heart attacks (lifting furniture & sandbagging) **During-flood:** 17/24 deaths male. Age range only reported: 8–78 years. Causes: 9 drowned (1 boat related, 2 car related, entered barricaded area, 1 slipped off embankment, & 1 child played near swollen stream), 2 heart attacks (evacuating), 3 lightning related (1 in car hit by tree struck by lightning; 1 in house burned after lightning strike, 1 struck by lightning while cleaning metal milking cans), 1 in car crash (avoiding flooded river involved another car). **Post-flood:** 4 heart attacks (cleaning up flooded basements), 1 asphyxiation (gas pump use in basement), 1 electrocution (used pump in flooded basement).
Smith, Young *et al.* 2013 [50] Australia	Case reports Queensland floods Dec 2010–Jan 2011 Cases of leptospirosis (and other flood-related infections) in flood-affected communities	Standard notification case reporting and usual laboratory surveillance, plus enhanced surveillance through health service providers. Surveyed cases on residential history 1 month prior to onset of illness (including temporary relocation due to flooding), consumption of food contaminated by floodwater; injuries (particularly breaches to skin related to flood exposure), contact with animals; and exact details of exposure to floodwater and involvement in flood recovery.	**During/Post-flood:** Nine confirmed leptospirosis cases were associated with floodwater. All of the cases of leptospirosis were: ill within 2 to 30 days, from floods events, all were hospitalised, all male and the median age was 34 and all had direct exposure with floodwater and most had cuts or abrasions.
CCDR 2000 [51] Canada	Case report and cross sectional study Heavy rainfall leading to flooding of water into Canadian water supply 2000 Determines the scope, the likely cause, and the contributing factors of the outbreak of gastroenteritis in Walkerton, Ontario, in May and June 2000	The investigation comprised a descriptive study and a cross-sectional study. Intensive case-finding for the descriptive study identified 1,346 reported cases of gastroenteritis exposed to municipal water.	**Post-flood:** 1,304 of 1,346 reported *E. coli* cases were primary, 39 were secondary (exposed to a primary case and not to public water supply), and three were unclassified. 27 of 65 patients admitted to hospital developed hemolytic uremic syndrome. Six deaths were attributed to the outbreak. 57% of cases were female and the median age of cases was 29 years (range < 1 to 97 years). Several cases were prior to the floods (earliest April 15), the majority of cases were contracted between 16 and 26 May. Homes connected to and consuming public water supply, were 11.7 times more likely to develop gastroenteritis than those not exposed to public water supply. A dose response relationship with the risk of illness increasing with the quantity of water consumed was observed. Some residents continued to expose themselves to the water, despite the extensive publicity and a “boil water” advisory, via brushing teeth with the water and occasionally drinking it.

**Table A5:** During-flood risk factors identified from studies that examined risk factors for those flooded in terms of health effects (*i.e.*, answered research questions 1 and 3a). N.B. ↑ = risk factor; ↓ = protective factor; − = not significant; [x] indicates study reference number.

	Mortality	Gastro illness	Mental illness	Physical illness	Injuries
**Gender** **(M= male; ** **F= female)**	− [17,20] M 10–29↑ [16] M 35–54↑ [36] M↑ [16,36]	F↑ [18]	− [34]	− [34]	− [18]
**Age**	− [20] 10–29↑ [16,35] 30–59↓ [16] >55↑ [17] >60↑ [16] >70↑ [35]	− [18]	>60↑ [34]	>60↑[34]	− [18,19]
**Ethnicity**	− [17]				
**Tenure**			− [34]	− [34]	
**Flooding indoors**		− [18]			↑ [18]
**Clean up involvement**		↑ [18]			↑ [18]
**Skin exposure to water**		↑ [18]			↑ [18]
**Exposed food**		− [18]			
**Private pond water supply**		↑ [18]			
**Public water supply**		− [18]			
**Tank water**		− [18]			
**Location (distance to flood)**			− [34]	− [34]	
**Vehicle occupancy**	↑ [20]				
**Used car to evacuate**	↓ [20]				
**Used car for other reason**	↑ [20]				
**High blood alcohol content **	↑ [20] (not comparative)				

**Table A6:** Post-flood risk factors identified from studies that examined risk factors for those flooded in terms of health effects (*i.e.*, answered research questions 1 and 3a). N.B. ↑ = risk factor; ↓ = protective factor; − = not significant; [x] indicates study reference number.

	Physical illness	Mental illness	PTS	Injuries	Respiratory illness	Gastro illness	Health care use
Age	<45↑ [30] − [31] older age ↑[28]	>60− [22,32] younger age↑ [31] 55–64↑ [31] older age ↑[28]	<65↑[22] older age↑[27]	older age −[28]	<15− [29] >65− [29]	increasing age↑ [24]	
Gender	M↑ [30] − [28,31]	F↑[22] M↑[31] −[28,32]	F↑[22,27]	−[28]	− [29]	− [24]	M↑ [25]
Married	↑[30]	↓[32]					
Lower education	− [31] ↓[30]	− [32]	↑[27]				
Lower SES	− [31] ↑[28] ↓[30]	↑[28,31,32]		↑[28]	↑[29]		
Existing health/ previous symptoms		↑[22,32] − [31,33]	↑[22]			↑[21]	
Access to health care	↓[28]	↓[28]		− [28]			
Medication interruption	↑[41]						
Non-US citizen	↑[28]	↑[28]		− [28]	↑[29]		
Greater local language proficiency	↑[28]	↑[28]		− [28]	↑[29]		
Ethnicity (Hispanic)	↑[28]	−[28]		− [28]			
Foreign born	− [28]	↓[28]		− [28]			
Mold exposure					↑[29]		
Family conflict					↑[29]		
Non-smoker	↑[30]						
Non-drinker	↑[30]						
Existing chronic GI						↑[21]	
Public water supply						−[21]	
Drinking water dose response						− [21]	
Direct floodwater contact						↑[21]	
Indirect floodwater contact						↑[21]	
Adverse event from flooding/ trauma	↑[28]	↑[28]	↑[27]	↑[28]			
Flooding to home/property	− [28]	− [28] ↑[22]	↑[22]	↑[28]		↑[21]	
Problems with insurance		↑[22]	↑[22]				
Uninsured		↑[22]	−[22]				
Evacuation		↑[22]	↑[22]				
Prolonged recovery/ trauma consequences		↑[22]	↑[22,26]				
Less warning time		↑[22]	↑[22]				
Rental housing		↑[22]	− [22]				
Water depth		− [22]	↑[22]				
Vulnerable housing		− [22]	↑[22]				
Decreasing distance from flood						↑[24]	
Personality trait:							
Briskness			↓[26]				
Perseveration			↑[26]				
Sensory sensitivity			− [26] ↓@15 months [26]				
Endurance			↓[26]				
Emotional reactivity			↑[26]				
Activity			↓@15 months; − @3 months & 3 yrs [26]				
